# On the risks of secure attachment in infancy: Childhood irritability and adolescent depression predicted by secure attachment and high approach behaviours at 14-months towards a caregiver living with inter-parental violence

**DOI:** 10.3389/frcha.2023.1143125

**Published:** 2023-04-17

**Authors:** Jonathan Hill, Nicky Wright, Helen Sharp, Andrew Pickles, Howard Steele

**Affiliations:** ^1^ School of Psychology and Clinical Language Sciences, University of Reading, Reading, United Kingdom; ^2^ Department of Psychology, Manchester Metropolitan University, Manchester, United Kingdom; ^3^ Institute of Population Health, University of Liverpool, Liverpool, United Kingdom; ^4^ Department of Biostatistics & Health Informatics, King’s College, London, United Kingdom; ^5^ The New School for Social Research, New School, New York, NY, United States

**Keywords:** attachment, strange situation procedure, interparental violence exposure, irritability, adolescent depression, inter-partner violence

## Abstract

**Introduction:**

The secure infant turns to a caregiver for comfort when distressed or threatened. Does this level of openness create vulnerability where the caregiver is unsupportive or aggressive? In this study we examined prospectively whether secure attachment in infancy, and approach behaviours on reunion with a parent, were associated with childhood emotional dysregulation (irritability) and adolescent depression among children exposed to inter-parental violence.

**Methods:**

We followed 219 families recruited from the general population during pregnancy (members of the Wirral Child Health and Development Study; WCHADS), with attachment assessments (Strange Situation Procedure; SSP) at 14 months, maternal interviews about inter-parental violence at 2.5, 5 and 7 years, and parent and teacher rated irritability at 9 years (CBCL). At age 13 years, 199 young people rated their levels of depression (SMFQ). In addition to the standard SSP classification, a latent variable reflecting approach behaviours during reunions was generated from the SSP dimensional scores and a factor score extracted. Analyses used path analysis using the gsem command in Stata.

**Results:**

There were interactions between attachment security and inter-parental violence for age 9 irritability (*p* = .084) and age 13 depression (*p* = .039) which arose from greater levels of psychopathology among secure children exposed to inter-parental violence. Similarly, higher approach behaviours during SSP reunions among children exposed to inter-parental violence were associated with irritability (interaction term *p* = .004) and depression (interaction term *p* < .001). Among children who were not exposed to partner violence higher approach behaviours in the SSP were associated with lower irritability and depression.

**Conclusion:**

Infant behaviours characteristic of attachment security in the Strange Situation Procedure may not equip children to deal with exposure to inter-parental violence and associated parental negativity.

## Introduction

Secure attachment has been conceptualised as good expectations on the infant's part, and trust in the caregiver and the wider world. This is consistent with evidence of robust links between security of attachment to mother and later social competence with peers (1). Groh et al. (1) assembled meta-analytic evidence from 80 independent samples and 4,441 children, reporting a small to moderate effect size of secure attachment (*d* = .39). These effects were unrelated to age of child suggestive of continuity of maternal sensitive responsiveness, in these mostly low-risk families. But what of those circumstances where infants who have developed a secure attachment subsequently experience adverse family conditions?

As long ago as 2002, Belsky et al. (2) argued that although the case can be made that secure attachment will confer resilience in the face of adversity, “…we would be remiss if we did not observe that reasoned arguments could be made for just the opposite predictions. For example, only when ecological conditions are supportive of development will early security predict enhanced functioning in the future” (2). Independent evidence that secure attachment may represent risk came from the pioneering study of maternal depression undertaken by Radke-Yarrow et al. (3) in the 1980s which showed that the children most resilient at 4-year follow-up were the children who had been insecure-avoidant with their depressed mothers. Those who had been secure, had the most problematic behaviour later (3). Further evidence that secure attachment may confer risk in the presence of unsupportive ecological conditions comes from a study of maternal and child anxiety disorders (4). Maternal social anxiety disorder was strongly associated with child internalising symptoms at ages 4–5 years, but only among children who had been rated secure in the Strange Situation Procedure (SSP) at 14 months. Similarly mothers’ higher interpretation of threat when reading a story with their children was associated with child anxiety disorder, but only in secure children.

The possibility that secure attachment confers risk in the presence of inter-parental violence (IPV) and conflict has not previously been examined. Inter-parental violence and conflict pose major threats to children's emotional and physical welfare and are associated with emotional and behavioural problems in children. Studies dealing with genetic confounds have demonstrated environmental effects (5, 6). Some of these effects are mediated *via* the parent-child relationship. A parent who is in a hostile and conflicted partner relationship is likely to be more hostile and aggressive toward their children and less sensitive and emotionally responsive to their children's needs (7). In addition to an impact on the parents’ behaviours with their children, conflict and violence in the home are likely to be frightening for a young child, and to pose fundamental questions as to whether their parents are people they can turn to for their own needs (8). This in turn raises a question regarding the way children cope in such a threatening family environment. On the one hand this may be through the preservation of a supportive relationship with a parent, in spite of the conflict (9) or with a sibling (10). On the other, the child may have to find their own individual coping strategies in regulating emotions and finding sources of relief from fear, anger or sadness (11). Davies et al.'s (11) Emotional Security Theory (EST) brings out the complexity and challenge for the child. In their operationalisation of the theory, less adaptive patterns of responding include “mobilizing” comprising high levels of vigilance and emotional arousal together with continued attempts to gain comfort from parents, “dominant” in which the threat is countered by the child through attempts to control, sometimes aggressively, the parental behaviours, and “demobilizing” responses such as freezing or withdrawal. Their characterisation of the “secure” pattern is of particular relevance to the study described here. It is conceptualised as reflecting the child's, “…underlying confidence that interparental disputes will be effectively managed and regulated in a way that maintains family harmony” (11). Behaviourally this is evidenced in only brief expressions of distress, and a limited concern for parents. Davies et al. (11) have reported promising findings showing associations between the secure pattern and better child adjustment in the face of inter-parental violence and conflict.

This detailed and sophisticated conceptual and empirical framework helps to bring out key questions in relation to parent-child attachment in the presence of high levels of within family threat. First the secure pattern of responding is thought to be characterised by a degree of disengagement from the conflict, in contrast to the behavioural and emotional engagement of the child with the parent which characterises the “secure” infant in the SSP (12). Second, the framework focuses on the child's adaptation to the parental conflict and not the child's orientation to the parent when themselves worried, anxious or sad, which is the focus of the assignment of the infant to an attachment category. Based on the EST we might predict that the “secure” pattern of behaviours would entail a degree of caution in expressing distress with, and seeking comfort from, a parent. In other words the “secure” organisation of behaviours seen in the SSP, may be different from the “secure” organisation of behaviours of the child shown towards parents in conflict with each other. Similarly at the representational level, if security in the SSP reflects trust in the caregiver, and confidence that they can provide relief in the face of threat, this may be poorly matched to the reality of the child whose parents are physically aggressive towards each other. On the other hand, if early secure attachment reflects a capacity to revise interpretations and expectations in the light of experience, then it may be associated with a subsequent revision of the child's representation of the trustworthiness of their parents’ caregiving. This would be consistent with evidence that adverse experiences during childhood may lead to a change from early secure to later insecure attachment, referred to as “lawful discontinuity” (13).

Most commonly the SSP is used to assign infants to secure and insecure categories based on rater judgements of a range of infant behaviours mainly at the second reunion. The strength of this method is that it reflects a combination of the infant's approach behaviours with the caregiver and the effectiveness of the reunion in leading to a reduction in distress. However dimensional ratings are also made in the SSP of specific behaviours which, if they were to persist during childhood, may be expected to put children exposed to parental violence and conflict at risk (proximity seeking and contact maintenance) or confer protection (avoidance). While the question of whether or not a dimensional characterisation of attachment has advantages over categorical assignments has been debated for a long time (14), these behavioural dimensions have received relatively little attention. However a recent study using multimodal measures of attachment-related behaviour such as dyadic contact duration and infant velocity of approach toward the mother, showed that these detailed observations were strongly associated with the SSP dimensions, supporting their validity (15).

Establishing targets for early intervention requires evidence of associations between early child or family characteristics and later psychopathology. The promotion of secure attachment has been a common target for early intervention, and so addressing questions discussed earlier regarding attachment security in the presence of inter-parental violence and discord, in relation to mental health outcomes in the long term is important (16). Similarly more needs to be known about the long term consequences of early exposure to inter-parental violence (17).

Depression in adolescence is common, with a prevalence of 23.4% in girls and 8.6% in boys (18). It is a major source of suffering and impairment, and commonly recurs through adolescence and into adult life. Identifying targets for early intervention to reduce adolescent depression is therefore a key goal. There are probably several pathways to adolescent depression, one of which is *via* childhood irritability (19–21). Irritability is commonly identified as a subset of externalizing behaviours characterised by a low threshold for getting angry, and frequent or intense angry responses. In view of the evidence for an environmental effect of inter-parental violence on childhood externalising behaviours, this may represent a pathway from exposure to domestic violence to adolescent depression. In this study we therefore examined both child irritability at age 9 years and depression at age 13 years. As outlined earlier directional hypotheses for the role of early attachment, could be based on risk arising where a child shows attachment behaviours towards physically aggressive parents, or on resilience arising from a child's ability to revise their representations based on experience. For this study, given that the SSP is behavioural rather than representational, we hypothesised that secure attachment and high approach behaviours in the SSP would be associated with increased child irritability and adolescent depression specifically in children exposed to inter-parental violence.

## Materials and methods

### Procedure and sample

The participants were members of the Wirral Child Health and Development Study (WCHADS), a prospective epidemiological longitudinal study in the North West of England, United Kingdom. It was designed to investigate vulnerability and resilience in the face of environmental stressors and their role in child and adolescent psychopathology. Families were recruited in pregnancy and followed over several assessment points during infancy up to when the children were mean age 12.88 (s.d. = 0.53) years (referred to as “age 13”). This uses a two-stage stratified design in which a consecutive general population sample (the “extensive” sample) is used to generate a smaller “intensive” sample stratified by psychosocial risk (psychological abuse in the partner relationship) and both are followed in tandem. The extensive sample was identified from consecutive first-time mothers who booked for antenatal care at 12 weeks gestation between 12 February 2007 and 29 October 2008. The booking clinic was administered by the Wirral University Teaching Hospital which was the sole provider of universal prenatal care on the Wirral Peninsula, a geographical area bounded on three sides by water. Socio-economic conditions on the Wirral range between the deprived inner-city and affluent suburbs, but with very low numbers from ethnic minorities.

The study was introduced to the women at 12 weeks of pregnancy by clinic midwives who asked for their agreement to be approached by study research midwives when they attended for ultrasound scanning at 20 weeks gestation. Ethical approval for the study was granted on four occasions covering the assessment points reported here, by the Cheshire North and West Research Ethics Committee on 27 June 2006 (reference no. 05/Q1506/107), 7th June 2010 (reference no. 10/H1010/4) and on 22nd December 2014 and 8th June 2020 (reference no. 14/NW/1484). At each assessment point written informed consent was obtained before administration of measures. Of those approached by study midwives, 68.4% gave consent and completed the measures, yielding an “extensive” sample of 1,233 mothers (mean age = 26.8 years, S.D. = 5.8 years, range 18–51 years) with surviving singleton babies who were available for postnatal follow-up. In the extensive sample 41.8% were in the most deprived quintile of UK neighbourhoods (22), consistent with high levels of deprivation in some parts of the Wirral. A total of 48 women in the extensive sample (3.9%) described themselves as other than white British. Details of recruitment to the study are shown in Sharp et al. (23). Maternal responses to questions about psychological abuse in their current or recent partner relationship (23, 24) were used to generate the stratified “intensive” sample of mothers and children for more detailed study. These intensive sample children were assessed at two prenatal time points, and postnatally on eleven occasions up to age 13. Assessments for this study were made at 14 months (infant attachment), at 2.5, 5 and 7 years (inter-parental violence), 9 years (irritability), and at 13 years (depression).

Of the 316 recruited to the intensive sample at 32 weeks gestation, 268 mothers and children attended for assessment of infant attachment status at 14 months. Of these, 238 mothers reported on inter-parental violence at three time points from age 2.5 to 7 years, data on age 9 years irritability were gathered from 219, and on age 13 depression from 199.

### Measures

#### Infant attachment status

Infant-mother attachment was assessed at 14 months using the Strange Situation Procedure (SSP) (12) in a purpose built room at the WCHADS centre, with filming through a one-way mirror and with cameras in the room. The SSP is a widely used laboratory procedure for the assessment of the attachment relationship between infants aged 12–20 months and their primary caregiver. The procedure was designed to capture the balance of the activation of the attachment and exploratory systems under conditions of increasing stress. The full assessment consists of 8 episodes, which involve a standardized sequence of separations and reunions between infant and mother (12). Five 7-point scales are rated to assess the infant's attachment behaviour during the reunion episodes: (1) Proximity seeking; (2) Contact maintenance; (3) Resistance to interaction; (4) Avoidance of contact and (5) Disorganization. Following the protocol outlined in Ainsworth et al. (12), infants are classified in three “organised” groups, as insecure-avoidant (A), secure (B), or insecure-resistant (C). Disorganized attachment (D) was coded using the Maine and Solomon criteria (25). For these analyses the secure-insecure contrast was used. The proximity seeking, contact maintenance and avoidance of contact scales were used to generate an approach factor as described in the “Statistical analyses” section.

All assessments of infant-mother attachment were rated by reliable, trained coders in Steele's attachment research lab. In the current sample, one trained rater who was blind to all other study data coded all infant-mother SSPs. To evaluate inter-rater reliability, 53 assessments (20%) were selected randomly for coding by a second trained rater who was also blind to the study details. The two coders achieved inter-rater reliability for both three-way (87% exact agreement; kappa = 0.79) and four-way classification (81% exact agreement; kappa = 0.72) coding schemes, and for the behavioural dimensions all ICCs ≥ .80.

#### Inter-parental violence

Inter-parental violence (IPV) was measured using the Partner Conflict Calendar (26) administered at the age 2.5 years, age 5 and age 7 interviews with mothers. The respondent is presented with a prompt card which displays a series of violent acts and is asked to report whether any of these have occurred between themselves and their partner, and if so, provide the date at which it occurred. Responses provided were categorically coded for the presence of IPV, with a single presence/absence (0/1) variable reflecting whether or not there had been an episode of violence between the 14 months assessment of attachment status and age 7 years.

#### Irritability age 9 years

Maternal reports of child symptoms were obtained at 9 years using the Child Behaviour Checklist (CBCL), which has been extensively employed in studies of child and adolescent emotional and behavioural disorders. It has 99 items each scored 0 (not true), 1 (somewhat or sometimes true), and 2 (very true or often true). Teacher reports of child symptoms were obtained using the Teacher Report Form (TRF), also a widely used measure of child and adolescent symptomatology. Where both parent and teacher scores were available (*N* = 201) the highest value was used in data analyses. If only one report from either the parent (*N* = 13) or the teacher (*N* = 5) was available, then that was used as the outcome variable. The ODD (oppositional defiant disorder) dimension of irritability was generated based on the items previously identified by confirmatory factor analyses (CFA) in adolescents (27), and on CFA with data collected at ages 2.5, 3.5 and 5.0 years in this study (28). Cronbach's alpha for parent and teacher irritability scales were .73 and .78.

#### Depression age 13 years

Adolescent depression was assessed using child report on the Short Mood and Feelings Questionnaire (SMFQ), which includes 13 items assessing DSM depression symptoms over the prior 2 weeks. The SMFQ is a widely used self-report measure which has been validated against other depression questionnaires and diagnostic interviews (29).

#### Confounds

Socioeconomic status was determined using the revised English Index of Multiple Deprivation (IMD) (22) based on data collected from the UK Census in 2001. According to this system, postcode areas in England are ranked from most deprived (i.e., IMD of 1) to least deprived (i.e., IMD of 32,482) based on neighbourhood deprivation in seven domains: income, employment, health, education and training, barriers to housing and services, living environment, and crime. All mothers were given IMD ranks according to the postcode of the area where they lived and assigned to a quintile based on the UK distribution of deprivation. Age of mother and the age they left full time education were recorded at the first assessment. Child sex was recorded at birth.

### Statistical analyses

Bivariate associations were examined using Spearman's correlations. All analyses were conducted in Stata version 17 (30). Child irritability and depression scores were highly skewed with a mode of zero so not suitable for transformation. Several of the SSP scores were also highly skewed. Therefore, the gsem command in Stata was used to generate the approach factor score and to test the main study hypotheses, with scores treated as ordinal or count variables. Confirmatory factor analysis was conducted in gsem on the six SSP variables treated as ordinal scores to generate a factor representing approach-avoidance behaviours in the SSP. A latent variable factor score was extracted for analysis. The study hypotheses were tested in path analysis using the gsem command in Stata with scores modelled as Poisson counts. All models included the covariates, variables reflecting stratification, main effects of attachment/approach variables and violence, and the interaction terms between attachment/approach and violence. Variables were standardised prior to generating interaction terms. Separate models were estimated for age 9 irritability and age 13 depression symptom outcomes, and for attachment classification and approach score. Interactions were plotted using the margins command in Stata, showing the association between approach score and child outcome in violence exposed and unexposed groups.

## Results

The factor loadings for the six SSP scores on the single approach latent variable are shown in Supplementary Table S1. A factor score was extracted for all subsequent analyses. Of the 219 children included in the analyses of irritability at age 9 years, 106 (48%) had been rated secure in the SSP at 14 months, and 50 (23%) were exposed to inter-parental violence between the ages of 14 months and 7 years. There was no association between the rate of secure attachment in the unexposed (83/169 = 49%) and in the exposed (22/50 = 44%) groups, c^2^ = 0.334, *p* = .563). In Table 1 showing bivariate associations among study variables it can be seen that there was a small association between attachment security and approach to the caregiver in the reunions in the SSP.

**Table 1:** Bivariate associations (Spearman's rho) among study variables.

	Attachment security	Approach factor	IPV before child age 7	Left school ≤age 18	Deprivation	Child sex	Maternal age	Irritability child age 9
Approach	.145 (.031)							
IPV	−.038 (.578)	−.154 (.022)						
Left school ≤age 18	.021 (.765)	−.065 (.335)	.137 (.042)					
Deprivation	−.087 (.197)	.053 (.436)	.188 (.005)	.204 (.003)				
Child sex	.004 (.952)	.063 (.354)	.025 (.707)	.076 (.258)	.064 (.344)			
Maternal age	.058 (.407)	.060 (.372)	−.186 (.005)	−.292 (<.001)	−.300 (<.001)	.041 (.546)		
Irritability age 9	−.005 (.946)	−.116 (.085)	.181 (.007)	.143 (.034)	.021 (.751)	−.019 (.784)	−.142 (.035)	
Depression age 13	.007 (.918)	−.088 (.590)	.008 (.914)	.023 (.747)	.083 (.246)	.353 (<.001)	.083 (.246)	.259 (<.001)

For all independent variables and for age 9 irritability *N* = 219, for age 13 depression *N* = 199. Values of *p* are shown in brackets. IPV, interparental violence.

Comparison of mean approach scores in the secure (*N* = 106), avoidant (*N* = 23), resistant (*N* = 19) and disorganized (*N* = 71) groups highlighted the variability in approach behaviours in the insecure group. In pairwise comparisons of mean approach scores in one-way ANOVA (model F 3,215 = 27.747, *p* < .001) the avoidant group mean was lower than the secure group mean (*p* < .001) and the disorganised group mean (*p* < .001), but the resistant group mean was higher than the secure (*p* = .008) and the disorganized (*p* < .001). The difference in approach behaviours between secure and disorganized groups was small and non-significant. Thus the secure-insecure demarcation does not provide a discriminating measure of approach behaviours on reunion in the SSP. Also shown in Table 1 there were small, but statistically significant associations between inter-parental violence and socio-economic deprivation, leaving school at age 18 or younger, and young maternal age at the birth of the first child, illustrating co-occurrence of family risks. Attachment security was not associated with age 9 irritability nor with age 13 depression. Higher approach behaviours in the SSP were associated with lower irritability at age 9 but not with young adolescent depression.

**Figure 1:**
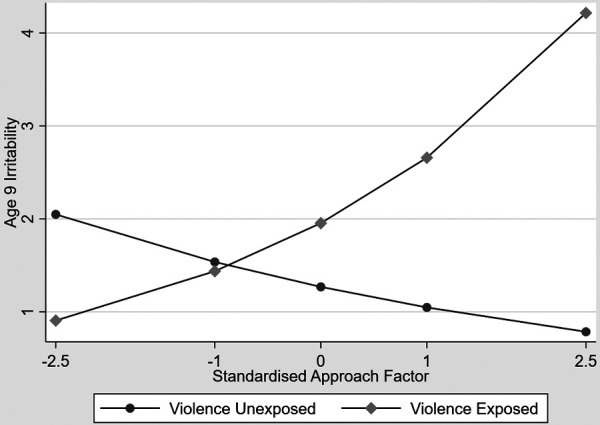
Associations between increasing approach and age 9 irritability contrasting children exposed to interparental violence and unexposed children.

After accounting for the planned confounders, deprivation, leaving school at 18 or under, maternal age at first child, child sex, and variables reflecting the sample stratification there was an attachment security by exposure to partner violence interaction in the prediction of age 9 irritability (unstandardised estimate = .45 95% CI −.06 to.97, *p* = .086). There was also an approach to caregivers by exposure to partner violence interaction (unstandardised estimate = .50, 95% CI .16 to.84, *p* = .004) which is shown in Figure 1. Increasing approach was associated with increasing irritability in exposed children and with decreasing irritability in the unexposed group. The plots represent linear effects of log symptom scales.

**Figure 2:**
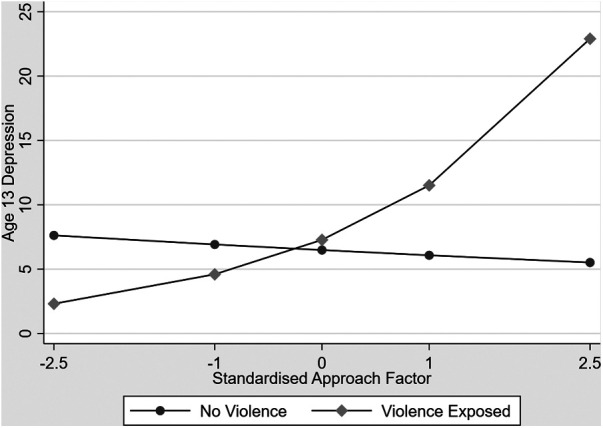
Associations between increasing approach and age 13 depression contrasting children exposed to interparental violence and unexposed children.

There was an attachment security by exposure to partner violence interaction in the prediction of age 13 depression (unstandardised estimate = .30, 95 CI .01 to .59, *p* = .039). There was also an approach to caregivers by exposure to inter-parental violence interaction in the prediction of age 13 depression (unstandardised estimate = .52, 95% CI .32 to .73, *p* < .001) which is shown in Figure 2.

We examined the size of the effects reflecting vulnerability associated with secure attachment and high approach behaviours in children exposed to inter-parental violence, and reflecting a protective role for secure attachment and high approach behaviours in children brought up without exposure up to age 7 years. This was done by comparing the proportional differences in symptoms in the attachment groups, either secure compared with insecure, or approach behaviours 1.s.d. above the mean compared with 1.s.d below the mean. The smaller exposed (*N* = 50) compared to unexposed (*N* = 169) groups accounted for substantial differences in 95% confidence intervals in the two groups. In each of the four analyses, with secure-insecure and approach-avoidance, age 9 irritability and age 13 depression, there was a greater vulnerability effect of secure or high approach in the exposed children, than protective effect in unexposed children. In line with the models for the interaction terms, this was more evident for approach/avoidance than for secure/insecure.

In the exposed group the proportional increase in irritability associated with secure attachment was 38% (95% CI −24% to 99%) while the proportional decrease in the unexposed group was 12% (95% CI −36% to 11%). In analyses using the approach-avoidance variable both the proportional increase associated with high approach (85%, 95% CI −31% to 201%) in the exposed group and the proportional decrease in the unexposed group (32%, 95% CI −50% to −14%) were greater than for the secure-insecure comparison.

For age 13 depression in the exposed group the proportional increase in depressive symptoms associated with secure attachment was 29% (95% CI −5% to 63%), while the proportional decrease in the unexposed group was 4% (95% CI −16% to 7%). By contrast in analyses using the approach factor, in the exposed group the proportional increase in depressive symptoms associated with high approach was much larger than for secure attachment, 150% (95% CI 53% to 247%), while the proportional decrease in the unexposed group was 12% (95% CI −23% to −2%), also larger than for secure attachment.

## Discussion

Based on our hypothesis that the emotional and behavioural openness of the secure infant will increase their vulnerability to the conflict and hostility commonly found in homes with inter-parental violence, we predicted that the combination of secure attachment and exposure to partner violence will be associated with higher levels of later dysregulated emotions and depressive symptoms than in unexposed children. This prediction was supported in analyses of attachment security in the SSP at 14 months, and of parent and teacher ratings of child irritability at age 9 years, and adolescent self-reported depression at age 13 years. We also hypothesised that the vulnerability may arise specifically from the child's tendency to approach their parent when distressed or threatened in spite of the negative environment of a home with inter-parental violence. We found that this tendency is not tracked precisely by attachment security. While as expected, approach scores based on SSP dimensions during the reunions were higher in the secure than the insecure groups, they were lower in the insecure-avoidant infants, but higher in the insecure-resistant infants, compared to those with secure attachment. We therefore hypothesised that increasing approach behaviours seen in the SSP would provide a more specific index of vulnerability than attachment security and so would show a stronger moderator effect with inter-parental violence. Using a high approach-low avoidance factor generated from the six SSP reunion dimensions, we found that children with high approach scores exposed to interparental violence, had elevated irritability and depression scores, and this was in marked contrast to children who had not been exposed to inter-parental violence. The strength of associations using the approach factor were somewhat greater than those for the secure-insecure difference, suggesting that the vulnerability may arise specifically from the tendency to seek comfort from a parent in a violent relationship.

Our findings add further to the evidence that children exposed to inter-parental conflict and violence face a formidable challenge in finding adaptive responses. As Davies and Martin (11) have indicated, the adaptation may entail a degree of emotional and behavioural caution, very different from the openness of the secure infant in the SSP. As we outlined earlier we do not know whether, behaviourally there is homotypic continuity of attachment behaviours in the face of major adversity, whereby children seek comfort even in the face of parental negativity, or heterotypic continuity with attachment security evidenced in changing behaviours. We have described evidence consistent with there being a pathway characterised by persistence of attachment behaviours even in the face of inter-parental violence, creating child and adolescent vulnerability.

Our findings bear on the broader question of whether there is an association between attachment security assessed in the SSP and later emotion dysregulation and depression as a main effect. Meta-analyses have reported small effect sizes for of attachment security and internalising symptoms but these are difficult to interpret in relation to this question because of considerable heterogeneity in sampling, attachment measures, ages of children, and cross-sectional vs. longitudinal design (31). The extent of this heterogeneity is highlighted in a recent meta-analysis in which associations between attachment security and adolescent depression were weaker in longitudinal than cross-sectional designs, for males than for females, and for attachment measures other than questionnaires (32). Once these were accounted there was a statistically significant, but very small (*b* = .047) association between insecure attachment and adolescent depression. Taken together, the findings of the meta-analyses and the evidence presented here imply that at least in respect of attachment status assessed behaviourally in the SSP, there are context dependent, rather than main effects of attachment security on child and adolescent emotion dysregulation and depression.

The marked differences in approach behaviours on reunion between the resistant and avoidant infants suggested that for investigations of the developmental implications of attachment behaviours the insecure category is a blunt tool. This was to some degree supported by our findings. For both the age 9 and age 13 years outcomes, while the pattern of findings were the same for the secure/insecure categories and the approach factor, the effects were somewhat stronger with the latter. There are wider implications of two kinds. First the existing coding system for the SSP includes a dimensional richness and specificity which has rarely been explored and which could enable novel developmental questions to be examined using data which are readily available. Second the dimensional approach to the attachment relationship lends itself to a more complex characterisation than the categorical approach permits. This is exemplified in studies which view attachment as a multidimensional control system accounting for exploratory as well as approach behaviours, and parental as well as infant behaviours (33).

The strengths of the study include the prospective measurement over substantial periods of time, using a range of methods, observational, interview, and parental, teacher and self-report questionnaire. The sample was recruited from the general population in a defined geographical area and stratified sampling allowed us to use time intensive observations with a subsample of known elevated psychosocial risk. Associations between younger age at first pregnancy, higher deprivation, IPV, and childhood irritability, were consistent with available evidence for co-occurrence of these risks and poor outcomes (34), supporting generalisability from this sample. Ratings of the SSP were made blind both to subsequent exposure to inter-parental violence and child and adolescent mental health outcomes. However statistical power for two-way interactions was limited by the size of the group of children exposed to inter-parental violence further divided into two roughly equal groups according to attachment status. In view of the associated risks of Type 2 errors reported values of “p” and the range of 95% confidence intervals may be less important than the pattern of findings which was very similar across two indices of attachment status, and across theoretically related outcomes at time points separated by four years.

The implications of the findings are far reaching. They imply that secure attachment, and the propensity to display attachment behaviours, are not straightforwardly positive or advantageous. Rather they contribute to development in a context dependent manner. Furthermore, in the extremely negative family environment of inter-parental violence, behaviours which under other circumstances are positive, can create vulnerability. This clearly requires further investigation, of the reproducibility of the findings, and also of the role of attachment assessed using different methods at different ages, and of other adverse family environments. The possibility has to be considered that interventions which aim to promote secure attachment in infants and young children, without assessing and aiming to reduce inter-parental violence or discord, may inadvertently increase their vulnerability to later emotional and behavioural problems.

## Data Availability

Due to ethical constraints supporting data cannot be made openly available. Supporting data are available to bona fide researchers on approval of an application for access. Further information about the data and conditions for access are available at the University of Liverpool Research Data Catalogue: https://doi.org/10.17638/datacat.liverpool.ac.uk/564.

## References

[1] Groh AM Fearon RP Bakermans-Kranenburg MJ van IJzendoorn MH Steele RD Roisman GI. The significance of attachment security for children’s social competence with peers: a meta-analytic study. Attach Hum Dev. (2014) 16:103–36. 10.1080/14616734.2014.883636 24547936 PMC4021853

[2] Belsky J Fearon RP. Infant-mother attachment security, contextual risk, and early development: a moderational analysis. Dev Psychopathol. (2002) 14:293–310. 10.1017/s0954579402002067 12030693

[3] Radke-Yarrow M Richters J Wilson WE. Child development in a network of relationships. In: Hinde RA Stevenson-Hinde J, editors. Relationships within families: Mutual influences. Oxford: Clarendon Press (1988). p. 48–67.

[4] Murray L Pella JE De Pascalis L Arteche A Pass L Percy R Socially anxious mothers’ narratives to their children and their relation to child representations and adjustment. Dev Psychopathol. (2014) 26:1531–46. 10.1017/S0954579414001187 25422977

[5] Jaffee SR Moffitt TE Caspi A Taylor A Arseneault L. Influence of adult domestic violence on children's internalizing and externalizing problems: an environmentally informative twin study. J Am Acad Child Adolesc Psychiatry. (2002) 41:1095–103. 10.1097/00004583-200209000-00010 12218431

[6] Harold GT Sellers R. Annual research review: interparental conflict and youth psychopathology: an evidence review and practice focused update. J Child Psych Psychiatry. (2018) 59:374–402. 10.1111/jcpp.12893 29574737

[7] Sherrill RB Lochman JE DeCoster J Stromeyer SL. Spillover between interparental conflict and parent–child conflict within and across days. J Fam Psychol. (2017) 31:900. 10.1037/fam0000332 28594199 PMC5662492

[8] Harold GT Leve LD Sellers R. How can genetically informed research help inform the next generation of interparental and parenting interventions? Child Dev. (2017) 88:446–58. 10.1111/cdev.12742 28160281 PMC5567989

[9] Neighbors B Forehand R McVicar D. Resilient adolescents and interparental conflict. Am J Orthopsychiatry. (1993) 63:462–71. 10.1037/h0079442 8372913

[10] Davies PT Parry LQ Bascoe SM Martin MJ Cummings EM. Children’s vulnerability to interparental conflict: the protective role of sibling relationship quality. Child Dev. (2019) 90:2118–34. 10.1111/cdev.13078 29916198 PMC6301125

[11] Davies PT Martin MJ Sturge-Apple ML Ripple MT Cicchetti D. The distinctive sequelae of children’s coping with interparental conflict: testing the reformulated emotional security theory. Dev Psychol. (2016) 52:1646. 10.1037/dev0000170 27598256 PMC5048543

[12] Ainsworth MDS Blehar MC Waters E Wall SN. Patterns of attachment: A psychological study of the strange situation. New York: Psychology Press (2015). 466.

[13] Weinfield NS Sroufe LA Egeland B. Attachment from infancy to early adulthood in a high-risk sample: continuity, discontinuity, and their correlates. Child Dev. (2000) 71:695–702. 10.1111/1467-8624.00178 10953936

[14] Fraley RC Spieker SJ. Are infant attachment patterns continuously or categorically distributed? A taxometric analysis of strange situation behavior. Dev Psychol. (2003) 39:387. 10.1037/0012-1649.39.3.387 12760508

[15] Prince EB Ciptadi A Tao Y Rozga A Martin KB Rehg J Continuous measurement of attachment behavior: a multimodal view of the strange situation procedure. Infant Behav Dev. (2021) 63:101565. 10.1016/j.infbeh.2021.101565 33887566

[16] Wright B Edginton E. Evidence-based parenting interventions to promote secure attachment: findings from a systematic review and meta-analysis. Glob Pediatr Health. (2016) 3:1–14. 10.1177/2333794X16661888 PMC4995667 27583298

[17] LeMoult K Humphreys KL Tracy A Hoffmeister JA Ip E Gotlib IH. Meta-analysis: exposure to early life stress and risk for depression in childhood and adolescence. J Am Acad Child Adolesc Psychiatry. (2020) 59:842–55. 10.1016/j.jaac.2019.10.011 31676392 PMC11826385

[18] Daly M. Prevalence of depression among adolescents in the US from 2009 to 2019: analysis of trends by sex, race/ethnicity, and income. J Adolesc Health. (2022) 70:496–99. 10.1016/j.jadohealth.2021.08.026 34663534

[19] Eyre O Hughes RA Thapar AK Leibenluft E Stringaris A Davey Smith G Childhood neurodevelopmental difficulties and risk of adolescent depression: the role of irritability. J Child Psych Psychiatry. (2019) 60:866–74. 10.1111/jcpp.13053 PMC6767365 30908655

[20] Whelan YM Leibenluft E Stringaris A Barker ED. Pathways from maternal depressive symptoms to adolescent depressive symptoms: the unique contribution of irritability symptoms. J Child Psychol Psychiatry. (2015) 56:1092–100. 10.1111/jcpp.12395 25665134 PMC4855627

[21] Sorcher LK Goldstein BL Finsaas MC Carlson GA Klein DN Dougherty LR. Preschool irritability predicts adolescent psychopathology and functional impairment: a 12-year prospective study. J Am Acad Child Adolesc Psychiatry. (2022) 61:554–64. 10.1016/j.jaac.2021.08.016 34481916 PMC9951107

[22] Noble M Wright G Dibben C Smith G McLennan D Anttila C Report to the office of the deputy prime minister: the english indices of deprivation 2004 (revised). London (UK): Neighbourhood Renewal Unit (2004).

[23] Sharp H Pickles A Meaney M Marshall K Tibu F Hill J. Frequency of infant stroking reported by mothers moderates the effect of prenatal depression on infant behavioural and physiological outcomes. PloS One. (2012) 7(10):e45446. 10.1371/journal.pone.0045446 23091594 PMC3473033

[24] Main M Solomon J. Procedures for identifying infants as disorganised/disoriented during the ainsworth strange situation. In: Greenberg MT Cicchetti D Cummings EM, editors. Attachment in the preschool years. Chicago, IL: University of Chicago Press (1990). p. 121–60.

[25] Moffitt TE Caspi A Krueger RF Magdol L Margolin G Silva PA Do partners agree about abuse in their relationship?: a psychometric evaluation of interpartner agreement. Psychol Assess. (1997) 9:47. 10.1037/1040-3590.9.1.47

[26] Ehrensaft MK Moffit TE Caspi A. Clinically abusive relationships in an unselected birth cohort: men’s and women’s participation and developmental antecedents. J Abnorm Psychol. (2004) 113:258. 10.1037/0021-843X.113.2.258 15122946

[27] Stringaris A Zavos H Leibenluft E Maughan B Eley TC. Adolescent irritability: phenotypic associations and genetic links with depressed mood. Am J Psychiatry. (2012) 169:47–57. 10.1176/appi.ajp.2011.10101549 22193524 PMC3660701

[28] Vidal-Ribas P Pickles A Tibu F Sharp H Hill J. Sex differences in the associations between vagal reactivity and oppositional defiant disorder symptoms. J Child Psych Psychiatry. (2017) 58:988–97. 10.1111/jcpp.12750 PMC5575540 28573761

[29] Angold A Costello EJ Messer SC Pickles A. Development of a short questionnaire for use in epidemiological studies of depression in children and adolescents. Int J Methods Psychiatr Res. (1995) 5:237–49.

[30] StataCorp. Stata statistical software. Version 17. College Station, TX, StatCorp LLC (2021).

[31] Groh AM Roisman GI van IJzendoorn MH Bakermans-Kranenburg MJ Fearon RP. The significance of insecure and disorganized attachment for children’s internalizing symptoms: a meta-analytic study. Child Dev. (2012) 83:591–610. 10.1111/j.1467-8624.2011.01711.x 22235928

[32] Spruit A Goos L Weenink K Rodenburg R Niemeyer H Stams G The relation between attachment and depression in children and adolescents: a multilevel meta-analysis. Clin Child Fam Psychol Rev. (2020) 23:54–69. 10.1007/s10567-019-00299-9 31392452 PMC7000490

[33] Gagliardi M. Human attachment as a multi-dimensional control system: a computational implementation. Front Psychiatry. (2022) 13:844012. 10.3389/fpsyg.2022.844012 PMC9521434 36186275

[34] Fergusson DM Woodward LJ. Maternal age and educational and psychosocial outcomes in early adulthood. J Child Psychol Psychiatry. (1999) 40(3):479–89. 10.1111/1469-7610.00464 10190348

