# Supplementary material for: On the risks of secure attachment in infancy: Childhood irritability and adolescent depression predicted by secure attachment and high approach behaviours at 14-months towards a caregiver living with inter-parental violence
Source: Front Child Adolesc Psychiatry. 2023 Apr 17;2:1143125. doi: 10.3389/frcha.2023.1143125 (PMC11748798; doi:10.3389/frcha.2023.1143125)
Supplement: Supplementary file 1 [file Table1.docx]

Supplementary Table 1: Factor loadings for six SSP scores on a single approach latent variable

|  | Unstandardized Estimate | Std err | P value |
| --- | --- | --- | --- |
| Proximity seeking 1 | 1.31 | .09 | <.001 |
| Proximity seeking 2 | .76 | .06 | <.001 |
| Contact maintenance 1 | 2.20 | .18 | <.001 |
| Contact maintenance 2 | .86 | .07 | <.001 |
| Avoidance 1 | -.55 | .05 | <.001 |
| Avoidance 2 | -.83 | .07 | <.001 |
